# Preventive Effect of Crocin on Osteoporosis in an Ovariectomized Rat Model

**DOI:** 10.1155/2014/825181

**Published:** 2014-08-14

**Authors:** Peng-Chong Cao, Wen-Xing Xiao, Ya-Bo Yan, Xiong Zhao, Shuai Liu, Jing Feng, Wei Zhang, Jun Wang, Ya-Fei Feng, Wei Lei

**Affiliations:** ^1^Department of Orthopaedics, Xijing Hospital, The Fourth Military Medical University, No. 127, West Changle Road, Xi'an, Shaanxi 710032, China; ^2^Department of Orthopaedics, Baoji Central Hospital, No. 8, Jiangtan Road, Baoji, Shaanxi 721008, China

## Abstract

The purpose of this study was to investigate the therapeutic effects of crocin on ovariectomy-induced osteoporosis in rats. Female Sprague-Dawley rats were randomly assigned to a sham-operated group (sham) and five ovariectomy (OVX) subgroups, that is, OVX with vehicle (OVX), OVX with 17*β*-estradiol (*E*_2_, 25 *μ*g/kg/day), and OVX with graded crocin doses (5, 10, or 20 mg/kg/day). Daily oral administration of *E*_2_ or crocin started 4 weeks after OVX and lasted for 16 weeks. Our results showed that crocin dose-dependently inhibited the BMD reduction of L4 vertebrae and femurs caused by OVX and prevented the deterioration of trabecular microarchitecture, which were accompanied by a significant decrease in skeletal remodeling as evidenced by the lower levels of bone turnover markers. Furthermore, crocin reversed the oxidative stress status in both serum and bone tissue. The present study indicates that the administration of crocin at higher doses over a 16-week period can prevent OVX-induced osteoporosis in rats without hyperplastic effects on the uterus, which may, at least partially, be attributed to crocin's antioxidative property. In brief, crocin is a natural alternative for postmenopausal osteoporosis treatment in elderly women.

## 1. Introduction

Osteoporosis is a systemic skeletal disease characterized by reduced bone mass and microarchitectural deterioration of bone tissue with a consequent increase in bone fragility and susceptibility to fractures [1]. According to data released by the World Health Organization (WHO), osteoporosis affects approximately 75 million people throughout Europe, the USA, and Japan [2]. The incidence of osteoporosis increases dramatically with life expectancy. Accordingly, the risk of osteoporotic fractures and their associated costs is rising rapidly due to population aging [3]. In the elderly, hip fractures are closely associated with mortality [4]. Hormone deficiency is known to impair cancellous metaphyseal bone and reduce BMD in humans and animals; therefore, the estrogen deficiency in postmenopausal women has been regarded as a critical cause of this population's susceptibility to osteoporosis [5]. Osteoporosis is twice as common in women as in men [6], and approximately one in three women over 50 years old experiences an osteoporotic fracture in her lifetime [7].

Clinically, hormone replacement therapy (HRT) has been a popular therapeutic strategy designed for postmenopausal osteoporosis [8, 9]. However, the long-term application of HRT has potential malignant effects on reproductive tissues [10–13]. Other medicines that stimulate bone formation (e.g., growth hormone, sodium fluoride, and parathyroid hormone) or inhibit bone resorption (e.g., bisphosphonates and calcitonin) may prevent bone loss progression in established osteoporosis. However, these medications are not affordable for a large proportion of the world population, especially in developing countries, and these medications have side effects, such as gastrointestinal reactions, cancers, osteonecrosis of the jaw, and reduced skeletal strength [14–17]. Consequently, to substitute or reduce the medicines used currently, there are efforts to develop new drugs with improved therapeutic efficacy, fewer undesirable side effects, and lower price.

Through thousands of years of human experimentation, belief in the safety, efficacy, and economy of “natural” products has contributed to these products' widespread clinical application in relieving women's postmenopausal symptoms. In recent decades, Chinese medicinal herbal extracts have been extensively investigated for their therapeutic effects on bone diseases and will undoubtedly continue to be used as a cost-effective alternative to commercial pharmaceutical products by traditional users [18–20]. In the search for new, naturally occurring antiosteoporosis agents, we identified saffron (*Crocus sativus* L.), which is a perennial herb of the iris family (Iridaceae) with a long history of use as a spice and food colorant in many countries, such as Iran, India, Greece, and China [21]. Saffron has been used in folk medicine for various purposes, as an antispasmodic, expectorant, stomachic, aphrodisiac, and emmenagogue [22, 23]. Crocin, a constituent of saffron, is a unique water-soluble carotenoid responsible for saffron's color and appears to possess various health-promoting properties [24–26]. Modern studies have demonstrated the pharmacological effects of saffron extracts and crocin, including antitumor [27], antidepression [28], antianxiety  [29], anti-inflammatory   [30, 31], memory enhancing [32], and cardioprotective effects as well as hypotensive activities [33, 34] and anticonvulsant ability [35]. Recent studies have also reported that crocin exerts protective effects against bone diseases, such as osteosarcoma and articular cartilage degeneration [36, 37].

A previous study by Assimopoulou et al. [38] showed that crocin exhibited significant radical scavenging activity and, thus, antioxidative activity, which was also confirmed by numerous in vivo studies [39, 40]. Crocin was also shown to revitalize cartilage and decrease bone deterioration along with inflammation and oxidative damage, which may be accredited to crocin's antioxidative nature [37]. Considering that recent studies have revealed the critical role of oxidative stress in the osteoporotic process [41], crocin's antioxidative properties may help improve osteoporosis; however, the effect of crocin on osteoporosis has not been investigated. Therefore, the aim of the present study was to systematically evaluate the effect of crocin on osteoporosis induced by OVX in rats and to further investigate the role of crocin's antioxidative activity in the extract's beneficial effects.

## 2. Materials and Methods

### 2.1. Crocin

Crocin was purchased from Sigma-Aldrich (product number 17304). General description is a highly unsaturated diterpene dicarboxylic acid ester found in saffron. Molecular mass is 976.3788. Molecular formula is C_44_H_64_O_24_. Property is light red brown powder. Related categories are cell biology, hematology, and histology. Purity is 98.96% determined by high performance liquid chromatography (HPLC) in the Research Center of Clinic Pharmacology and Pharmacy of the Fourth Military Medical University.

### 2.2. Animals and Treatments

Healthy three-month-old female Sprague-Dawley (SD) rats (wt. 226 ± 12 g) were obtained from the Experimental Animal Center of the Fourth Military Medical University (certificate number SCXK 2007-008). All the rats were adapted to laboratory environment for 1 week before the experiment. The acclimatized rats underwent either bilateral laparotomy (sham, *n* = 10) or bilateral ovariectomy (OVX, *n* = 50) as previously described [42]. Four weeks after recovering from surgery, the OVX rats were divided into 5 groups: OVX with vehicle (OVX, *n* = 10), OVX with 17*β*-estradiol (*E*_2_, oral, 25 *μ*g/kg/day, *n* = 10), OVX with a low crocin dose (L-C, oral, 5 mg/kg/day, *n* = 10), OVX with a medium crocin dose (M-C, oral, 10 mg/kg/day, *n* = 10), and OVX with a high crocin dose (H-C, oral, 20 mg/kg/day, *n* = 10). Treatment was initiated 4 weeks after OVX and lasted for 16 weeks. The body mass of each rat was monitored weekly, and the administered dose was adjusted accordingly.

Before being sacrificed, each rat was individually housed in a metabolic cage without food for 24 h. A urine sample was collected and acidified with 2 mL of 1 mol/L HCl. After laparotomy using anesthetized rats with pentobarbital sodium (intraperitoneal injection, 40 mg/kg), blood sample was collected via abdominal aorta puncture. Then, a serum specimen was harvested after centrifugation (2000 rpm for 20 min). Urine and serum samples were stored at −20°C until further testing and analysis. The uterus was removed; the wet weight was measured in grams, and the uterus index was calculated by dividing this weight by the body weight in kilograms. Femurs and the fourth lumbar (L4) vertebrae were dissected, wrapped with wet gauzes to prevent dehydration, and stored at −20°C for bone mineral density (BMD) measurement, trabecular microarchitecture analysis, and biomechanical testing.

### 2.3. BMD Measurement

The BMD of the L4 vertebrae and right femurs was estimated using dual-energy X-ray absorptiometry scanning (DEXA, GE Healthcare, USA) with small animal measurement software as reported previously [43]. The measurements were expressed as grams of mineral contents per cm^2^ of surface area. Scans were performed by the same blinded technician.

### 2.4. Three-Point Bending Test

Before mechanical testing, the left femurs were slowly thawed at room temperature. The length of each femur (distance from the intermalleolar to the intercondylar region) was measured with a micrometer, and the center of the diaphysis was determined. Then, the femurs were submitted to a three-point bending test using a material testing machine (MTS 858 Mini Bionix II, MTS Systems Corp., USA). Each femur was placed in the machine with two support points separated by a distance of 20 mm and loaded at a speed of 2 mm/min. In the movement of the central loading point, the load and displacement were recorded until the specimen was broken. Using the load-deformation curve, biomechanical parameters, including the maximum load, maximum stress, and Yong's modulus, were calculated according to previously described formulas [44].

### 2.5. Micro-CT Analysis

Based on the BMD, three representative L4 vertebrae from each group were selected to evaluate the trabecular microarchitecture using eXplore Locus SP preclinical specimen microcomputed tomography (Micro-CT, GE Healthcare, USA). Before the scans, the bones were positioned with gauze in the sample holder and allowed to reach room temperature. The L4 vertebrae were scanned from the anterior endplate in the posterior endplate direction (22 *μ*m/slice). The isotropic voxel resolution of bone was 22 *μ*m^3^. The volume of interest (VOI) was selected as a region 25 slices away from the anterior endplate to the posterior endplate, ranging to 125 slices. The three-dimensional images were reconstructed with the purpose of visualization and display. After analyzing the VOI, morphometric bone parameters, including bone volume over total volume (BV/TV), trabecular number (Tb.N), trabecular separation (Tb.Sp), trabecular thickness (Tb.Th), connectivity density (Conn.D), and structure model index (SMI), were obtained. The VOI analysis was performed blindly by the same operator.

### 2.6. Biochemical Analysis of Serum and Urine Specimens

The levels of serum calcium (S-Ca), serum phosphorus (S-P), serum alkaline phosphatase (ALP), urinary calcium (U-Ca), urinary phosphorus (U-P), and urinary creatinine (Cr) were measured on an automatic analyzer (Ciba-Corning 550, USA) using a diagnostic reagent kit. Serum osteocalcin (OC) concentration was determined using a rat OC ELISA kit (San Clemente, CA, USA).

### 2.7. Oxidative Stress Status Detection

Total SOD activity in serum was estimated by the method described by Kono [45]. One unit of SOD is defined as the amount of enzyme that causes 45% inhibition of NBT reduction under the assay conditions. As a marker of lipid peroxidation, the MDA level was assayed using the thiobarbituric acid (TBA) method described by Ohkawa et al. [46]. TBA reacts with MDA to form a diadduct, a pink chromogen, detectable at 532 nm. The source of reactive oxygen species (ROS) was analyzed by detecting the protein expression of manganese superoxide dismutase (MnSOD) and NADPH oxidase 4 (NOX4) by western blot.

### 2.8. Western Blot Analysis

The expressions MnSOD and NOX4 in bone tissue were determined by western blot analysis. Femurs were excised and cleaned of all muscles and connective tissue was removed. The bones were immediately frozen in liquid nitrogen and stored at −80°C until sample processing. Bone protein was extracted as previously described [47]. The lysate was centrifuged and the protein was quantified and separated by sodium dodecyl sulfate-polyacrylamide gel electrophoresis (SDS-PAGE) and then transferred onto a polyvinylidene difluoride (PVDF) membrane. After being blocked with 5% milk, the expressions of MnSOD and NOX4 were probed with specific antibodies (Cell Signaling, Santa Cruz, CA) overnight at 4°C, followed by incubation with the corresponding secondary antibodies at room temperature for 1 h. The blots were visualized with ECL-plus reagent (Amersham Pharmacia Biotech, USA). The MnSOD and NOX4 immunoblots were then stripped with strip buffer at 50°C for 30 minutes and reblotted for *β*-actin.

### 2.9. Statistical Analysis

The data are expressed as the mean ± SD. Statistical analysis was performed using one-way ANOVA combined with Bonferroni's multiple comparison test using SPSS 13.0. A *P* value <0.05 was considered significant.

## 3. Results

### 3.1. Body Weights and Uterine Index

**Figure 1:**
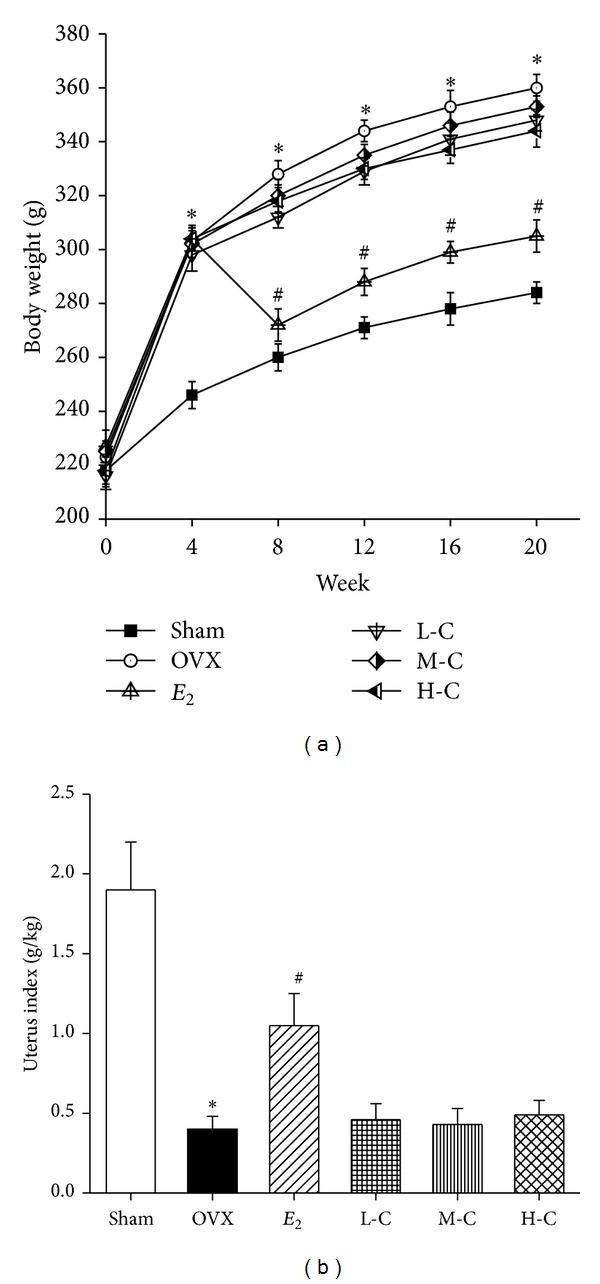
Effects of crocin or* E*_2_ on (a) body weight and (b) uterus index of OVX rats. Values are mean ± SD, *n* = 10. *P < 0.05 versus sham; ^**#**^ *P* < 0.05 versus OVX.

As shown in Figure 1(a), there were no significant differences in the initial mean body weight between the six study groups (all *P* > 0.05). At week 4, the OVX group was 23.2% heavier than the sham group (*P* < 0.05), while* E*_2_ significantly decreased the body weight to a level similar to that of the sham group 8 weeks after the operation (*P* < 0.05). However, none of the three crocin doses had a significant effect in preventing the body weight increase at any time point. As shown in Figure 1(b), the uterus weight in the OVX group was significantly reduced to 21.1% of the uterus weight in the sham group (*P* < 0.05), indicating the success of the surgical procedure.* E*_2_ significantly prevented uterine weight loss compared to the OVX group (*P* < 0.05). However, none of the crocin dose levels elicited any uterotrophic effects.

### 3.2. BMD of L4 Vertebrae and Femurs

**Figure 2:**
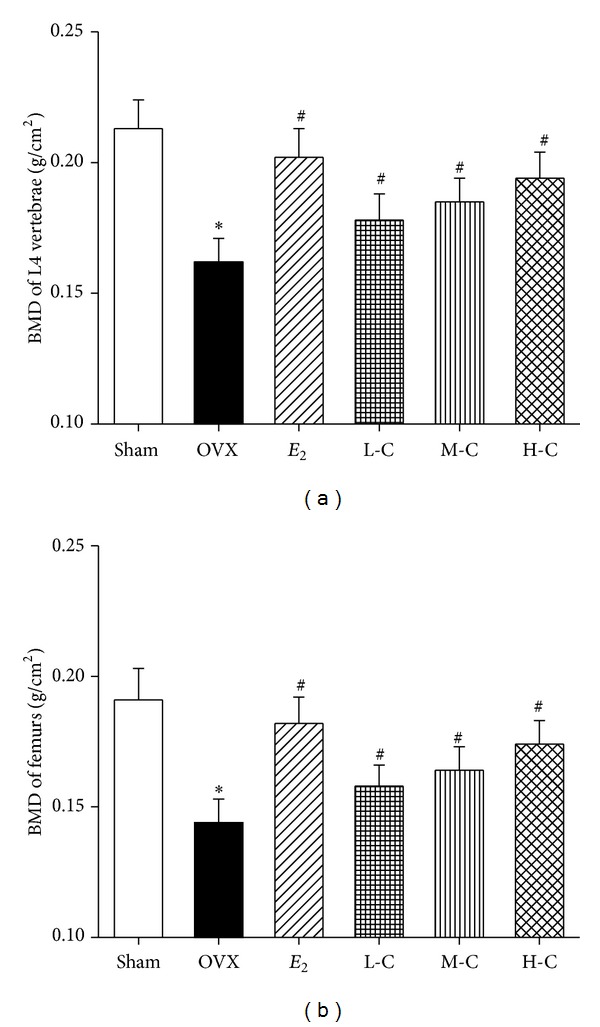
Effects of crocin or* E*_2_ on BMD of (a) L4 vertebrae and (b) right femurs of OVX rats. Values are mean ± SD, *n* = 10. *P < 0.05 versus sham; ^**#**^ *P* < 0.05 versus OVX.

The BMD of the L4 vertebrae and femurs is presented in Figure 2. These results demonstrate that OVX significantly decreased the BMD by 23.9% in the L4 vertebrae and by 25.6% in the femurs compared to the sham group (both *P* < 0.05). Compared to the OVX group, crocin treatment obviously prevented the BMD decrease in OVX-induced L4 vertebrae and femurs (all *P* < 0.05) in a dose-dependent manner.* E*_2_ also significantly increased the BMD of the L4 vertebrae and femurs (both *P* < 0.05), which was similar to that observed in the H-C crocin group (both *P* > 0.05).

### 3.3. Mechanical Testing of Femurs

**Figure 3:**
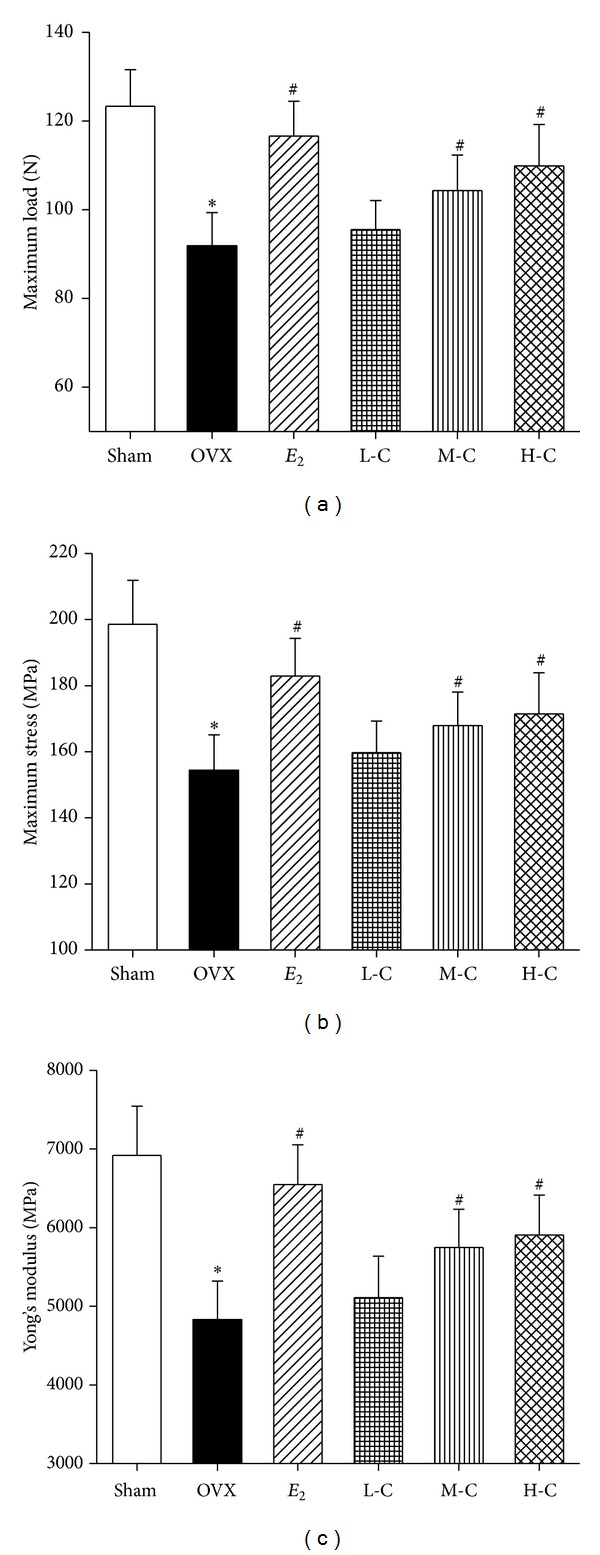
Effects of crocin or* E*_2_ on bone biomechanical parameters containing (a) maximum load, (b) maximum stress, and (c) Yong's modulus in femurs of OVX rats. Values are mean ± SD, *n* = 10. *P < 0.05 versus sham; ^**#**^ *P* < 0.05 versus OVX.

The results of the femur mechanical testing are presented in Figure 3. Compared with the sham group, 16 weeks of estrogen deficiency significantly decreased the maximum load by 25.5%, maximum stress by 22.3%, and Yong's modulus by 30.2% (all *P* < 0.05). Meanwhile, higher dosage crocin treatments (10 or 20 mg/kg/day) markedly prevented the OVX-induced tendency to decrease these parameters (all *P* < 0.05).* E*_2_ also increased the above-mentioned biomechanical parameters, which were significantly higher than those of the OVX group (all *P* < 0.05). It is worth noting that the increase in maximum load observed for the H-C crocin group was similar to that of* E*_2_ (*P* > 0.05).

### 3.4. Micro-CT Analysis of L4 Vertebrae

**Figure 4:**
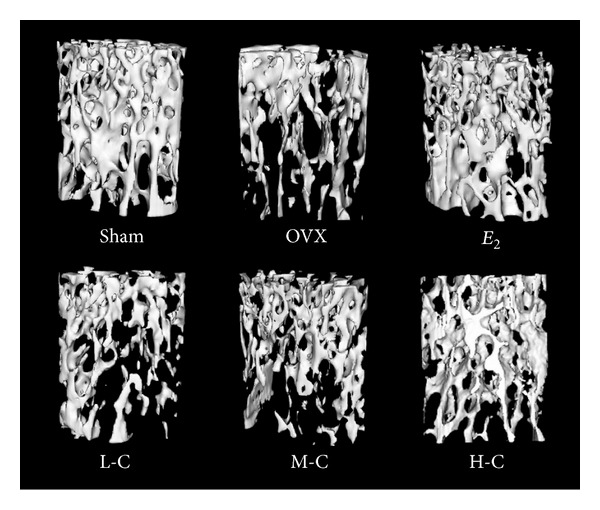
3D reconstruction images of VOI in L4 vertebrae showed the effects of crocin or* E*_2_ treatments on cancellous bone microarchitecture in OVX rats.

**Figure 5:**
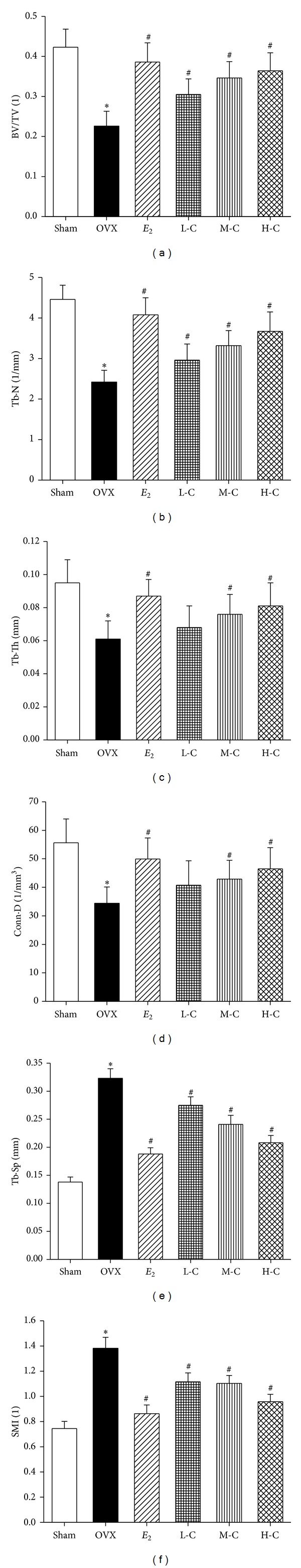
Effects of crocin or* E*_2_ on morphometric parameters including (a) BV/TV, (b) Tb.N, (c) Tb.Th, (d) Conn.D, (e) Tb.Sp, and (f) SMI in L4 vertebrae of OVX rats. Values are mean ± SD, *n* = 3. *P < 0.05 versus sham; ^**#**^ *P* < 0.05 versus OVX.

Three-dimensional reconstruction images of the L4 vertebrae showed differences in trabecular microarchitecture among the various treatment groups as represented in Figure 4. Analysis of the representative samples (Figure 5) indicated that OVX resulted in the deterioration of the trabecular bone microarchitecture, as demonstrated by the reduced BV/TV, Tb.N, Tb.Th, and Conn.D, compared with the sham group (all *P* < 0.05). In contrast, Tb.Sp and SMI were significantly increased in response to OVX compared to the sham group (both *P* < 0.05). Higher dosage crocin treatment (10 or 20 mg/kg/day) significantly improved the microarchitecture deterioration mentioned above (all *P* < 0.05), while* E*_2_ also reversed these parameters to the similar degree as that in the H-C group (all *P* > 0.05).

### 3.5. Biochemical Analysis of Serum and Urine Specimens

**Figure 6:**
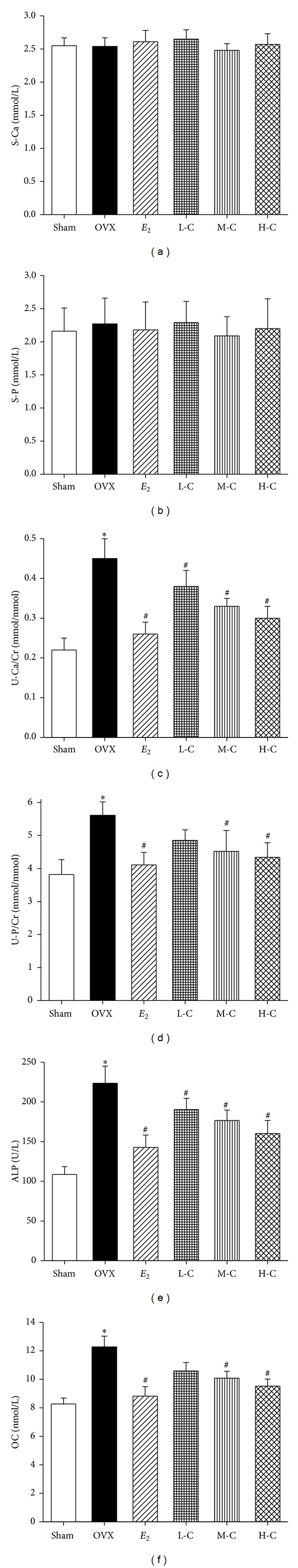
Effects of crocin or* E*_2_ on biochemical parameters including (a) S-Ca, (b) S-P, (c) U-Ca/Cr, (d) U-P/Cr, (e) ALP, and (f) OC in serum and urine of OVX rats. Values are mean ± SD, *n* = 10. *P < 0.05 versus sham; ^**#**^ *P* < 0.05 versus OVX.

Date presented in Figure 6 show the effects of crocin or* E*_2_ on biochemical parameters in the serum and urine of OVX rats. There were no significant differences in the values for S-Ca or S-P among all of the study groups (Figures 6(a)-6(b); both *P* > 0.05). However, the levels of U-Ca/Cr, U-P/Cr, ALP, and OC (Figures 6(c)–6(f)) were significantly increased in the OVX group by 104.5%, 36.9%, 105.9%, and 48.5% compared to the sham group (all *P* < 0.05), respectively. All three crocin doses significantly prevented the increases in U-Ca/Cr and ALP levels (all *P* < 0.05) in a dose-dependent manner. Higher dosage crocin treatment (10 or 20 mg/kg/day) significantly prevented the increases in U-P/Cr and OC levels (both *P* < 0.05). Again,* E*_2_ administration also reversed the above-mentioned increases, which were statistically significant. It is worth noting that the reductions in U-P/Cr, ALP, and OC in the H-C crocin group were similar to those observed for* E*_2_ (all *P* > 0.05).

### 3.6. Oxidative Stress Status in Serum and Bone Tissue

**Figure 7:**
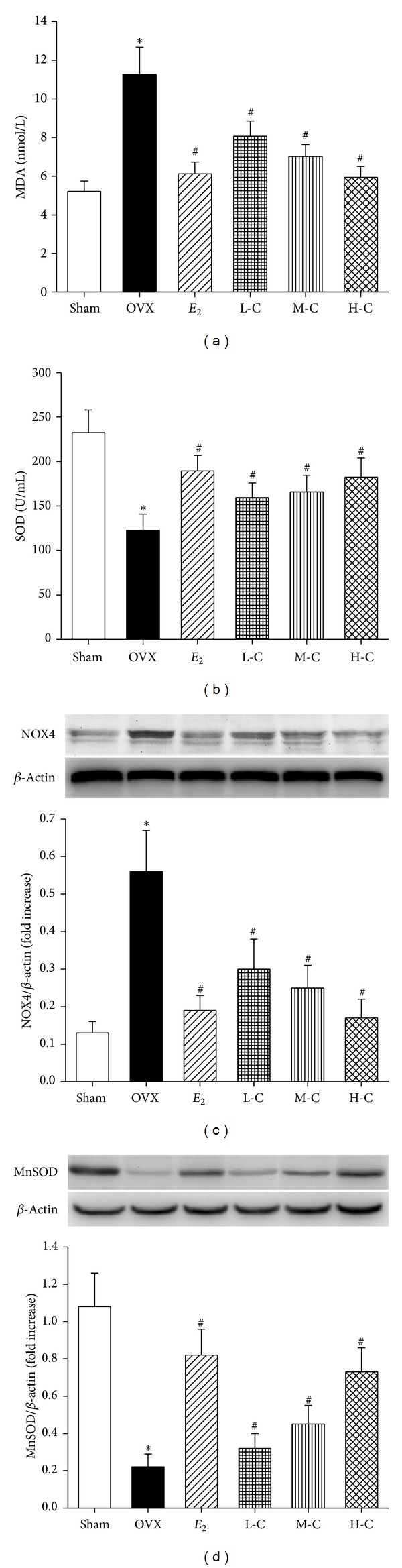
Effects of crocin or* E*_2_ on oxidative stress status including (a) MDA and (b) SOD in serum and (c) NOX4 and (d) MnSOD expression in bone tissue of OVX rats. Values are mean ± SD, *n* = 10. *P < 0.05 versus sham; ^**#**^ *P* < 0.05 versus OVX.

The serum ROS level and antioxidative statuses of the different experimental groups are presented in Figures 7(a)-7(b). The MDA level demonstrated a significant 116.1% increase compared to the sham group (*P* < 0.05). In contrast, the total SOD activity significantly decreased by 47.3% in response to OVX for 16 weeks compared to the sham group (*P* < 0.05). In order to further evaluate the potential sources of the radicals and the mitochondrial status, the protein expressions of NOX4 and MnSOD in bone tissue were detected using western blot. The data (Figures 7(c)–7(d)) showed that OVX caused significant increase in the expression of NOX4 and decrease in MnSOD compared with that in sham group (both *P* < 0.05). All crocin doses significantly decreased serum MDA content and bone NOX4 expression and stimulated SOD activity in both serum and bone tissue of OVX rats (all *P* < 0.05) in a dose-dependent manner.* E*_2_ also significantly exerted similar antioxidative effect to that of H-C crocin (all *P* > 0.05).

## 4. Discussion

Our study is the first to demonstrate the beneficial effects of crocin against OVX-induced osteoporosis in rats. The major findings of the present study are as follows: first, treatment with higher crocin doses significantly improved the bone mass, bone strength, bone microarchitecture, and bone turnover in OVX-induced osteoporotic rats; second, the highest crocin dosage exerted the best protective effect against osteoporosis, which was similar to that of* E*_2_; finally, crocin significantly attenuated oxidative stress in serum and bone tissue, indicating that crocin's beneficial effect on bone is attributed at least partially to crocin's antioxidative effects. Taken together, these results suggest the potential role of crocin as a natural alternative for postmenopausal osteoporosis management.

The high incidence, serious complications, financial burden, and dramatically decreased living quality indicate the severity of osteoporosis in humans. Despite the pharmacological and clinical advantages of HRT as a widely accepted therapeutic strategy for osteoporosis, serious side effects of long-term application have also been reported. Therefore, the development of new preventive and therapeutic drugs for osteoporosis is urgently needed. In recent decades, Chinese medicinal herbal extracts have been extensively investigated for their pharmacological effects related to bone protection. The commonly investigated herbal extracts, such as* Curcuma longa*,* Cistanche salsa*,* Acanthopanax senticosus*, and* Herba Epimedii* [48–51], have been reported to influence the proliferation and differentiation of osteoclasts (OCs) and osteoblasts (OBs) in vitro and/or have therapeutic potency in osteoporosis in vivo. This accumulation of evidence encouraged us to explore the use of other herbs and their potential therapeutic effects in osteoporosis.

Saffron is a well-known traditional Chinese medicine. Compared to saffron's other carotenoids, crocin possesses the highest coloring capacity; the carotenoid is known for quenching free radicals and is endowed with tumoricidal properties. Crocin has been shown to exhibit beneficial effects on many organs, including the nervous system (the most studied) and the gastrointestinal, cardiovascular, genital, endocrine, and immune systems [26]. Recent studies indicate that crocin can be used to manage bone diseases and can act as an efficient antiarthritic and antiosteosarcoma agent; however, crocin's role in the osteoporotic process was not clear from these studies. Hence, we systematically investigated whether crocin effectively improves impaired bone quantity and quality, thus exerting protective effects against osteoporosis in OVX rats.

Bone remodeling is the biologic process that mediates changes in the traits that influence bone strength. Any interruption in bone remodeling, such as menopause, will disturb the balance between formation and resorption and cause bone mass loss [52, 53]. Therefore, we used OVX rats as an animal model for human osteoporosis in vivo experiments. It has been reported that statistically significant bone loss can be seen after 30 days [54], so treatment was initiated 4 weeks after OVX. Consistent with other studies, OVX caused significantly higher body weights in our present study, which may be attributed to fat deposition caused by the lack of estrogen [55]. Previous studies suggest that estrogen plays an important role in stimulating the differentiation of progenitor cells through the osteoblast lineage but not the adipocyte lineage [56]. As expected, this excess body weight gain was completely prevented by* E*_2_ treatment. However, crocin at all dose levels did not prevent the increase in body weight induced by OVX and did not demonstrate any uterotrophic activity. The results suggest that crocin did not mimic the effects of* E*_2_ in the regulation of body weight and uterine tissue growth in the OVX rats.

Decreased BMD is one of the major factors jeopardizing bone strength, resulting in increased susceptibility to fractures  [57]. Thus, BMD measurement can best predict fracture risk [58]. The results in the present study showed that OVX reduced BMD in the right femurs and L4 vertebrae, which are rich in trabecular bone, while treatment with crocin dose-dependently prevented the decreases in BMD. Although BMD is among the strongest predictors of facture resistance, both empirical observations and theoretical analyses show that the biomechanical properties of bone and trabecular microarchitecture influence trabecular bone strength as well [59–61]. Three-point bending tests of the left femurs in our study indicated that the higher crocin doses (10 or 20 mg/kg/day) prevented the OVX-induced tendency toward decreased biomechanical parameters. Moreover, measurements of structural parameters using micro-CT also showed that treatment with crocin effectively restored the trabecular microarchitectural properties compared to the OVX group. In addition, the measurement of bone markers plays a role in osteoporosis diagnosis and treatment [62]. Bone mass loss, as evidenced by enhanced levels of ALP, OC, U-Ca/Cr, and U-P/Cr, indicated upregulation of bone turnover by OVX. The bone turnover markers above were dose-dependently reversed by crocin, indicating a reduction in bone turnover rate after treatment of crocin. Furthermore, it is also worth noting that crocin exhibits high efficacy and no major toxicity in experimental models [26], suggesting crocin's effectiveness and safety for treating osteoporosis in menopausal women.

Estrogen deficiency has been considered the seminal mechanism of osteoporosis in women, but epidemiological evidence in humans and recent mechanistic studies in rodents indicate that aging and the associated increase in ROS are the proximal culprits. In 2010, Manolagas reviewed the emerging evidence that provides a paradigm shift from the “estrogen-centric” account of involutional osteoporosis pathogenesis to one in which oxidative stress-related mechanisms intrinsic to bone are the protagonists [41]. Recently, the antioxidative properties of crocin have received increased attention. In the present study, crocin reversed the increase in ROS production (MDA) and improved the activity of the antioxidative enzyme SOD in both serum and bone tissue, thereby confirming crocin's antioxidative role in osteoporosis.

## 5. Conclusion

The present study demonstrated that daily oral administration of crocin over a 16-week period prevents estrogen deficiency-induced bone loss, inhibits the deterioration of trabecular microarchitecture, maintains the biomechanical competence of bone, decreases bone turnover rate, and does not stimulate an unwanted proliferation of the uterine tissues. Moreover, the antioxidative properties elicited by crocin may contribute to the compound's beneficial effect, rendering crocin a potent protector against oxidative stress-induced bone loss during osteoporosis. Our findings indicate that crocin has the potential to be further developed as a natural alternative for postmenopausal osteoporosis management in elderly women.

## References

[1] NIH Consensus Development Panel on Osteoporosis Prevention (2001). Osteoporosis prevention, diagnosis, and therapy. *The Journal of the American Medical Association*.

[2] Eastell R, Black DM, Boonen S (2009). Effect of once-yearly zoledronic acid five milligrams on fracture risk and change in femoral neck bone mineral density. *The Journal of Clinical Endocrinology & Metabolism*.

[3] Burge R, Dawson-Hughes B, Solomon DH, Wong JB, King A, Tosteson A (2007). Incidence and economic burden of osteoporosis-related fractures in the United States, 2005–2025. *Journal of Bone and Mineral Research*.

[4] Omsland TK, Emaus N, Tell GS (2014). Mortality following the first hip fracture in Norwegian women and men (1999–2008). A NOREPOS study. *Bone*.

[5] Marcus R (2002). An expanded overview of postmenopausal osteoporosis. *Journal of Musculoskeletal Neuronal Interactions*.

[6] Sugerman DT (2014). JAMA patient page. Osteoporosis. *JAMA*.

[7] Johnell O, Kanis JA (2006). An estimate of the worldwide prevalence and disability associated with osteoporotic fractures. *Osteoporosis International*.

[8] Stevenson JC (2005). Justification for the use of HRT in the long-term prevention of osteoporosis. *Maturitas*.

[9] Prelevic GM, Kocjan T, Markou A (2005). Hormone replacement therapy in postmenopausal women. *Minerva Endocrinologica*.

[10] Gray S (2003). Breast cancer and hormone-replacement therapy: the Million Women Study. *The Lancet*.

[11] Orija IB, Mehta A (2003). Hormone replacement therapy: current controversies. *Clinical Endocrinology*.

[12] Lacey JV, Mink PJ, Lubin JH (2002). Menopausal hormone replacement therapy and risk of ovarian cancer. *The Journal of the American Medical Association*.

[13] Rossouw JE, Anderson GL, Prentice RL (2002). Risks and benefits of estrogen plus progestin in healthy postmenopausal women: principal results from the Women’s Health Initiative randomized controlled trial. *The Journal of the American Medical Association*.

[14] Lee JK, Kim KW, Choi JY (2013). Bisphosphonates-related osteonecrosis of the jaw in Korea: a preliminary report. *Journal of the Korean Association of Oral and Maxillofacial Surgeons*.

[15] Riggs BL, Hodgson SF, O'Fallon MW (1990). Effect of fluoride treatment on the fracture rate in postmenopausal women with osteoporosis. *The New England Journal of Medicine*.

[16] (2013). In brief: cancer risk with salmon calcitonin. *Medical Letter on Drugs and Therapeutics*.

[17] Verhaar HJ, Lems WF (2009). PTH-analogs: comparable or different?. *Archives of Gerontology and Geriatrics*.

[18] Qi W, Yan Y-B, Lei W (2012). Prevention of disuse osteoporosis in rats by Cordyceps sinensis extract. *Osteoporosis International*.

[19] Zhao X, Wu ZX, Zhang Y (2011). Anti-osteoporosis activity of Cibotium barometz extract on ovariectomy-induced bone loss in rats. *Journal of Ethnopharmacology*.

[20] Zhang R, Liu ZG, Li C (2009). Du-Zhong (*Eucommia ulmoides* Oliv.) cortex extract prevent OVX-induced osteoporosis in rats. *Bone*.

[21] Hosseinzadeh H, Modaghegh MH, Saffari Z (2009). *Crocus sativus* L. (saffron) extract and its active constituents (crocin and safranal) on ischemia-reperfusion in rat skeletal muscle. *Evidence-Based Complementary and Alternative Medicine*.

[22] Rios JL, Recio MC, Ginger RM (1996). An update review of saffron and its active constituents. *Phytotherapy Research*.

[23] Zargari A (1990). *Medicinal Plants*.

[24] Hosseinzadeh H, Nassiri-Asl M (2013). Avicenna's (Ibn Sina) the canon of medicine and saffron (*Crocus sativus*): a review. *Phytotherapy Research*.

[25] Rezaee R, Hosseinzadeh H (2013). Safranal: from an aromatic natural product to a rewarding pharmacological agent. *Iranian Journal of Basic Medical Sciences*.

[26] Alavizadeh SH, Hosseinzadeh H (2014). Bioactivity assessment and toxicity of crocin: a comprehensive review. *Food and Chemical Toxicology*.

[27] Abdullaev FI, Espinosa-Aguirre JJ (2004). Biomedical properties of saffron and its potential use in cancer therapy and chemoprevention trials. *Cancer Detection and Prevention*.

[28] Wang Y, Han T, Zhu Y (2010). Antidepressant properties of bioactive fractions from the extract of *Crocus sativus* L.. *Journal of Natural Medicines*.

[29] Hosseinzadeh H, Noraei NB (2009). Anxiolytic and hypnotic effect of *Crocus sativus* aqueous extract and its constituents, crocin and safranal, in mice. *Phytotherapy Research*.

[30] Hong YJ, Yang KS (2013). Anti-inflammatory activities of crocetin derivatives from processed Gardenia jasminoides. *Archives of Pharmacal Research*.

[31] Nam KN, Park Y-M, Jung H-J (2010). Anti-inflammatory effects of crocin and crocetin in rat brain microglial cells. *European Journal of Pharmacology*.

[32] Hosseinzadeh H, Sadeghnia HR, Ghaeni FA, Motamedshariaty VS, Mohajeri SA (2012). Effects of saffron (*Crocus sativus* L.) and its active constituent, crocin, on recognition and spatial memory after chronic cerebral hypoperfusion in rats. *Phytotherapy Research*.

[33] Razavi M, Hosseinzadeh H, Abnous K, Motamedshariaty VS, Imenshahidi M (2013). Crocin restores hypotensive effect of subchronic administration of diazinon in rats. *Iranian Journal of Basic Medical Sciences*.

[34] Mehdizadeh R, Parizadeh M, Khooei A, Mehri S, Hosseinzadeh H (2013). Cardioprotective effect of saffron extract and safranal in isoproterenol-induced myocardial infarction in wistar rats. *Iranian Journal of Basic Medical Sciences*.

[35] Hosseinzadeh H, Talebzadeh F (2005). Anticonvulsant evaluation of safranal and crocin from *Crocus sativus* in mice. *Fitoterapia*.

[36] Li X, Huang T, Jiang G, Gong W, Qian H, Zou C (2013). Synergistic apoptotic effect of crocin and cisplatin on osteosarcoma cells via caspase induced apoptosis. *Toxicology Letters*.

[37] Hemshekhar M, Santhosh MS, Sunitha K (2012). A dietary colorant crocin mitigates arthritis and associated secondary complications by modulating cartilage deteriorating enzymes, inflammatory mediators and antioxidant status. *Biochimie*.

[38] Assimopoulou AN, Sinakos Z, Papageorgiou VP (2005). Radical scavenging activity of *Crocus sativus* L. extract and its bioactive constituents. *Phytotherapy Research*.

[39] El-Beshbishy HA, Hassan MH, Aly HAA, Doghish AS, Alghaithy AAA (2012). Crocin “saffron” protects against beryllium chloride toxicity in rats through diminution of oxidative stress and enhancing gene expression of antioxidant enzymes. *Ecotoxicology and Environmental Safety*.

[40] Asdaq SM, Inamdar MN (2010). Potential of Crocus sativus (saffron) and its constituent, crocin , as hypolipidemic and antioxidant in rats. *Applied Biochemistry and Biotechnology*.

[41] Manolagas SC (2010). From estrogen-centric to aging and oxidative stress: a revised perspective of the pathogenesis of osteoporosis. *Endocrine Reviews*.

[42] Li M, Shen Y, Wronski TJ (1997). Time course of femoral neck osteopenia in ovariectomized rats. *Bone*.

[43] Shen Y, Li Y-Q, Li S-P, Ma L, Ding L-J, Ji H (2010). Alleviation of ovariectomy-induced osteoporosis in rats by *Panax notoginseng* saponins. *Journal of Natural Medicines*.

[44] Turner CH, Burr DB (1993). Basic biomechanical measurements of bone: a tutorial. *Bone*.

[45] Kono Y (1978). Generation of superoxide radical during autoxidation of hydroxylamine and an assay for superoxide dismutase. *Archives of Biochemistry and Biophysics*.

[46] Ohkawa H, Ohishi N, Yagi K (1979). Assay for lipid peroxides in animal tissues by thiobarbituric acid reaction. *Analytical Biochemistry*.

[47] Schreiweis MA, Butler JP, Kulkarni NH (2007). A proteomic analysis of adult rat bone reveals the presence of cartilage/chondrocyte markers. *Journal of Cellular Biochemistry*.

[48] Wright LE, Frye JB, Timmermann BN, Funk JL (2010). Protection of trabecular bone in ovariectomized rats by turmeric (*Curcuma longa* L.) Is dependent on extract composition. *Journal of Agricultural and Food Chemistry*.

[49] Yamaguchi K, Shinohara C, Kojima S, Sodeoka M, Tsuji T (1999). (2E,6R)-8-hydroxy-2,6-dimethyl-2-octenoic acid, a novel Anti-osteoporotic monoterpene, isolated from Cistanche salsa. *Bioscience, Biotechnology and Biochemistry*.

[50] Hwang Y, Jeong I, Ahn KJ, Chung HY (2009). The effects of Acanthopanax senticosus extract on bone turnover and bone mineral density in Korean postmenopausal women. *Journal of Bone and Mineral Metabolism*.

[51] Zhang D, Zhang J, Fong C, Yao X, Yang M (2012). Herba epimedii flavonoids suppress osteoclastic differentiation and bone resorption by inducing G2/M arrest and apoptosis. *Biochimie*.

[52] Wronski TJ, Dann LM, Scott KS, Crooke LR (1989). Endocrine and pharmacological suppressors of bone turnover protect against osteopenia in ovariectomized rats. *Endocrinology*.

[53] Turner RT, Vandersteenhoven JJ, Bell NH (1987). The effects of ovariectomy and 17 beta-estradiol on cortical bone histomorphometry in growing rats. *Journal of Bone and Mineral Research*.

[54] Liu XQ, Cui L, Wu T (2005). Study of bone histomorphometric changes at regular intervals in OVX rats. *Chinese Journal of Osteoporosis*.

[55] McElroy JF, Wade GN (1987). Short- and long-term effects of ovariectomy on food intake, body weight, carcass composition, and brown adipose tissue in rats. *Physiology & Behavior*.

[56] Dang ZC, Van Bezooijen RL, Karperien M, Papapoulos SE, Löwik CWGM (2002). Exposure of KS483 cells to estrogen enhances osteogenesis and inhibits adipogenesis. *Journal of Bone and Mineral Research*.

[57] Park JA, Ha SK, Kang TH (2008). Protective effect of apigenin on ovariectomy-induced bone loss in rats. *Life Sciences*.

[58] Cummings SR, Bates D, Black DM (2002). Clinical use of bone densitometry: scientific review. *Journal of the American Medical Association*.

[59] Gibson LJ (1985). The mechanical behaviour of cancellous bone. *Journal of Biomechanics*.

[60] Rice JC, Cowin SC, Bowman JA (1988). On the dependence of the elasticity and strength of cancellous bone on apparent density. *Journal of Biomechanics*.

[61] Keaveny TM, Morgan EF, Niebur GL, Yeh OC (2001). Biomechanics of trabecular bone. *Annual Review of Biomedical Engineering*.

[62] Allende-Vigo MZ (2007). The use of biochemical markers of bone turnover in osteoporosis. *Puerto Rico Health Sciences Journal*.

